# Helicobacter Pylori and Celiac Disease Antibody Positivity Have a Higher Prevalence in Patients With Distal Gastrectomy: A Cross-Sectional Retrospective Study

**DOI:** 10.7759/cureus.42976

**Published:** 2023-08-05

**Authors:** Kubilay Issever, Ersin Kuloglu, Demet Sengul, Ali Muhtaroglu, Ahmet Cumhur Dulger

**Affiliations:** 1 Internal Medicine, Giresun University Faculty of Medicine, Giresun, TUR; 2 Pathology, Giresun University Faculty of Medicine, Giresun, TUR; 3 General Surgery, Giresun University Faculty of Medicine, Giresun, TUR; 4 Gastroenterology, Giresun University Faculty of Medicine, Giresun, TUR

**Keywords:** dyspepsia, antrectomy, celiac disease, helicobacter pylori, distal gastrectomy

## Abstract

Aims and objectives

Distal gastrectomy was a widely used therapeutic option for peptic ulcer and gastroesophageal reflux disease until quite recently. The consequences of anatomical and physiological changes following surgery in the gastric mucosa have been the object of interest for the scientist. In this study, we aimed to determine whether Helicobacter pylori (HP) infection and celiac disease were more common in patients with a history of distal gastrectomy.

Materials and methods

This is an observational retrospective study conducted at Giresun University Faculty of Medicine. The medical files of 35 patients with dyspepsia who had a history of distal gastrectomy for benign etiologies (antrectomy group) and 50 patients with dyspepsia (control group) were retrospectively analyzed.

Results

There were more males and older patients in the antrectomy group. Concerning the lab parameters, platelets, lymphocyte, and albumin levels were significantly lower, and urea, creatinine, anti-Endomisium Ig A (anti-EMA), and anti-tissue transglutaminase IgA (anti-tTGA) antibody positivity were significantly higher in the antrectomy group. Gastric biopsy results revealed a higher positivity of HP, atrophy, neutrophil, and lymphocytes in the antrectomy group. Correlation analysis revealed an inverse correlation between albumin and anti-EMA/atrophy positivity whereas a positive correlation between anti-EMA and HP/atrophy positivity.

Conclusions

HP infection and coeliac disease (CD) could be the problems that distal gastrectomy patients with dyspepsia can face during their follow-up. Concerning the pre-malignant potential of HP, its screening and eradication should be performed to prevent the malignant transformation of the remnant gastric tissue.

## Introduction

Distal gastrectomy, also called antrectomy, is a surgical procedure in which the distal third of the stomach (pyloric antrum) is resected either via laparoscopy or as an open surgery [1]. This procedure can be used to treat a wide range of gastric diseases from benign conditions such as gastric ulcers and gastric outlet obstructions to malignant or mortal conditions such as ulcer perforation, bleeding, and gastric neoplasms [2]. Reconstruction methods of this procedure that are widely used to establish gastrointestinal (GI) continuity are gastroduodenostomy (Billroth I), gastrojejunostomy (Billroth II), and Roux-en-Y gastrojejunostomy [1]. On the other hand, the discovery and wide usage of antacids and proton pump inhibitors reduced the high surgical rates caused by gastric ulcers in the late 20th century [3]. However, we have a burden consisting of many operated patients with some post-operative problems since this resection might change the normal function and natural environment of the gastric mucosa. Dumping syndrome, atrophic gastritis, recurrent ulcer disease, malabsorption syndromes, and early postoperative complications can be seen in the patients after this intervention [1,3]. 

Coeliac disease (CD) can present with digestive symptoms such as dyspepsia and bloating in its mild form while severe diarrhea, unexplained weight loss, malabsorption, and anemia can be seen in the severe form [4]. Diagnosis can be made with a detailed history and physical examination accompanied by autoantibodies (e.g., tissue transglutaminase 2-IgA, endomysial antibody Ig A), and upper GI endoscopy with duodenal biopsy [5]. Anti-tissue transglutaminase IgA (anti-tTGA) is the gold standard serological test for the diagnosis of CD, however, anti-endomysium Ig A (anti-EMA) testing may provide additional evidence for diagnosis, especially for patients with conflicting findings or weak Anti-tTGA positivity [6]. Duodenum is the first candidate tissue to be affected anatomically and physiologically by removing the distal stomach since it is adjacent to it. Since CD is an illness of the duodenal tissue, we can hypothesize that its prevalence might be changed in patients with antrectomy. However, there are few studies in the literature regarding the seroprevalence of coeliac auto-antibodies in dyspeptic patients with distal gastrectomy.

Helicobacter pylori (HP), which was first isolated from humans in 1982, is a curved, highly motile, gram-negative rod found in the mucus layer overlying the gastric mucosa [7]. Persons colonized with HP have higher gastrin levels, which are reduced by the eradication of the organism. This hyperacidity creates a predisposition to ulcerations and gastric neoplasms, especially in patients with persistent colonization [8]. As a result of this, HP can be attributed as one of the major causes of distal gastrectomies performed in the past 50 years. Moreover, persistent HP infection is one of the underlying causes of gastric stump cancer that might occur in some patients with distal gastrectomy [9]. Interestingly, fewer studies can be found in the literature regarding the prevalence of HP in patients with distal gastrectomy. This study aims to compare the laboratory, serologic, and gastric mucosa specimen findings of dyspeptic patients with and without distal gastrectomy.

## Materials and methods

Eighty-five patients who applied to the internal medicine and gastroenterology outpatient clinic of Giresun University Training and Research Hospital in the years 2021 and 2022 with complaints of dyspepsia were included in the study. Patient informed consent was not applicable due to the retrospective design of the study. Our study was approved by the Ordu University Clinical Research Ethical Committee (acceptance date: December 23, 2022, meeting number: 25, approval number: 292) as a retrospective study. All procedures performed in studies involving human participants were in accordance with the ethical standards of the institutional and/or national research committee and with the 1964 Helsinki Declaration and its later amendments or comparable ethical standards.

The inclusion criteria were being above 18, having dyspepsia as the cardinal symptom, being evaluated with upper GI endoscopy, and having a pathology result. The patients were divided into two groups according to their gastrectomy status. Thirty-five patients who underwent distal gastrectomy for at least a year were named as the “antrectomy group” whereas 50 patients who had no history of gastrectomy were determined as the “control group”. All patients in the antrectomy group had already undergone the same surgical procedure (antrectomy, bilateral truncal vagotomy, Billroth II gastroenterostomy) for the treatment of peptic ulcer and gastroesophageal reflux disease. The demographical, laboratory, and pathology results were obtained from the hospital data processing systems and analyzed retrospectively. Blood test results were the ones that were taken on the day of the upper GI endoscopy. Anti-tTGA and anti-EMA serology tests were also performed with the same blood sample. HP and other parameters of pathology specimens were studied by the same pathologist. HP, atrophy, neutrophil, lymphocyte, eosinophil, dysplasia, activity, and hyperemia were the parameters evaluated by the pathologist for the samples of the gastric mucosa. This was a retrospective study in which the eligible patients were selected randomly.

Data analysis was performed using Statistical Package for the Social Sciences (SPSS), version 26.0 (IBM Corp., Armonk, NY). The suitability of the numerical variables of the patients to the normal distribution was determined by looking at the skewness values. Except for glucose, creatine, and c-reactive protein (CRP) values, it was observed to comply with the rules of normal distribution. The reference value taken in the normal distribution was between ±1.5. The chi-square test was used to compare the essential characteristics, antibody, and pathology findings of patients who did not undergo gastrectomy and who underwent distal gastrectomy. Independent sample t-test or Mann-Whitney U test was used to compare laboratory parameters of patients who did not undergo gastrectomy and who underwent distal gastrectomy. Pearson or Spearman correlation tests were used to examine the relationship between distal gastrectomy patient status and laboratory, antibody, and pathology findings. Correlation coefficient; a relationship between 0.00-0.30 was considered as a low-level, between 0.30-0.70 as a medium-level, and between 0.70-1.00 as a high-level relationship. In the whole study, the significance levels were carried out by considering the values of 0.05 and 0.01.

## Results

Table 1 shows the demographical comparison between the groups. 65.7% of the antrectomy group was male while 70% of the control group was female and this gender difference was significant (p=0.002). The median age of the antrectomy group was 66,54±11,30 while it was 55,72±17,31 for the control group (p=0.001).

**Table 1:** The comparison of the groups in terms of age and gender *p<0,05, **p<0,01, 𝜒2: chi-square test (categorical data); t: independent sample t-test; Avg: average; S.D: standard deviation; Med: median; Min: minimum; Max: maximum

Demographical features		Control group (n:50)		Antrectomy group (n:35)		p
		n	%	N	%	
Gender	Female	35	70,0	12	34,3	0,002**
	Male	15	30,0	23	65,7	
Age	<60	30	60,0	12	34,3	0,035*
	≥60	20	40,0	23	65,7	
		Avg.±S.D Med. (Min.-Max.)		Avg.±S.D Med. (Min.-Max.)		
Age^t^		55,72±17,31 54 (16-84)		66,54±11,30 67 (43-92)		0,001**

In Table 2, we analyzed the lab test results of the groups. The antrectomy group was found to have significantly lower levels of platelets, lymphocytes, and albumin (p=0.026, 0.001, and <0.001, respectively); whereas, higher levels of urea and creatinine were detected for the antrectomy group (p=0.001 and 0.002, respectively). Analysis of the other parameters such as white blood cells, glucose, alanine aminotransferase (ALT), aspartate aminotransferase (AST), etc. revealed no significant difference between the groups. Patients with distal gastrectomy had significantly more positive anti-tTGA and anti-EMA results (p=0.013 and 0.009, respectively).

**Table 2:** The comparison of the groups in terms of lab parameters *p<0,05, **p<0,01, t: independent sample t-test; z: Mann-Whitney U test; Med: median; Min: minimum; Max: maximum; S.D: standard deviation; WBC: white blood cell; HGB: hemoglobin; HTC: hematocrit; MCV: mean corpuscular volume; PLT: platelets; LYM: lymphocyte; ALT: alanine aminotransferase; AST: aspartate aminotransferase; CRP: C-reactive protein; Anti-tTGA: anti-tissue transglutaminase antibody; Anti-EMA: anti-endomysium antibody

Parameters	Control group (n:50)	Antrectomy group (n:35)	p
	Med.±S.D.	Med.±S.D.	
WBC^t ^(10^6^/L)	6967,80±1898,05	6548,00±1265,76	0,257
HGB^t ^(g/dL)	12,35±2,26	11,71±1,74	0,165
HTC^t ^(%)	37,63±5,55	35,69±4,52	0,090
MCV^t ^(fL)	83,62±8,76	84,23±7,24	0,735
PLT^t ^(10^4^/ml)	274,64±80,92	238,20±59,68	0,026*
LYM^t ^(10^4^/ml)	2,08±0,51	1,65±0,62	0,001**
Glucose^z ^(mg/dL)	109,08±43,20 99,50 (52-345)	104,60±38,44 95,00 (59-260)	0,251
Urea^t ^(mg/dL)	29,18±8,93	37,91±15,36	0,001**
Creatinine^z ^(mg/dL)	0,73±0,19 0,70 (0,5-1,27)	1,01±0,62 0,90 (0,3-4)	0,002**
ALT^t ^(u/L)	17,62±9,57	18,11±6,53	0,792
AST^t ^(u/L)	18,94±5,38	21,60±7,25	0,056
Albumin (g/L)	45,20±4,05	39,59±6,40	0,000**
Sodium^t ^(mmol/L)	140,74±2,16	140,03±2,77	0,188
Potassium^t ^(mmol/L)	4,46±0,44	4,35±0,57	0,301
Calcium^t ^(mg/dL)	9,44±0,41	9,53±0,54	0,362
CRP^z ^(mg/L)	4,87±7,18 2,30 (0,1-38,6)	9,86±20,82 2,90 (0,2-109)	0,429
	n (%)	n (%)	
Anti-tTGA (-)	48 (96)	23 (65,7)	0,001**
Anti-tTGA (+)	2 (4)	12 (34,3)	
Anti-EMA (-)	48 (96)	26 (74,3)	0,009**
Anti-EMA (+)	2 (4)	9 (25,7)	

We compared the pathology results between the antrectomy and the control group; 48% of the control group and 77.1% of the antrectomy group got a positive HP result for their gastric biopsy specimen and this difference was also significant (p=0.003). Atrophy, lymphocyte, and neutrophil positivity were the other pathology parameters that were more commonly found in the antrectomy group (p=0.002, <0.001, and 0.016, respectively). Metaplasia, dysplasia, eosinophil, activity, and hyperemia positivity did not differ significantly between the groups (Table 3).

**Table 3:** The comparison of the groups in terms of pathology findings *p<0,05, **p<0,01,  𝜒2: chi-square test; H.pylori: Helicobacter pylori

Pathology Findings		Control group (n:50)		Antrectomy Group (n:35)		
		n	%	n	%	p
H. pylori	Negative	26	52,0	8	22,9	0,003**
	Slight positive	6	12,0	14	40,0	
	Moderate positive	6	12,0	8	22,9	
	Severe positive	12	24,0	5	14,3	
Metaplasia	Negative	41	82,0	23	65,7	0,145
	Slight positive	9	18,0	12	34,3	
Atrofi	Negative	48	96,0	24	68,6	0,002**
	Slight positive	2	4,0	11	31,4	
Neutrophil	Negative	22	44,0	3	8,6	0,000**
	Slight positive	6	12,0	15	42,9	
	Moderate positive	6	12,0	15	42,9	
	Severe positive	16	32,0	2	5,7	
Lymphocyte	Negative	14	28,0	1	2,9	0,016*
	Slight positive	11	22,0	11	31,4	
	Moderate positive	14	28,0	9	25,7	
	Severe positive	11	22,0	14	40,0	
Dysplasia	Negative	48	96,0	32	91,4	0,679
	Slight positive	2	4,0	3	8,6	
Eosinophil	Negative	34	68,0	30	85,7	0,148
	Slight positive	9	18,0	4	11,4	
	Moderate positive	7	14,0	1	2,9	
Activity	Negative	26	52,0	16	45,7	0,726
	Slight positive	24	48,0	19	54,3	
Hyperemia	Negative	24	48,0	14	40,0	0,611
	Slight positive	26	52,0	21	60,0	

Our final analysis was to determine the correlation between significant laboratory, coeliac serology, and pathology parameters. Correlation analysis revealed a slightly negative correlation between albumin and anti-EMA/atrophy positivity which means that lower albumin levels are associated with higher anti-EMA and atrophy positivity (p=0.037 and 0.001, respectively). In addition to that, anti-EMA positivity showed a slightly positive correlation with HP and atrophy positivity (p=0.022 and 0.038, respectively). As expected, anti-EMA showed a moderate correlation with anti-tTGA (p<0.001). Other parameters revealed no correlation between each other (Table 4).

**Table 4:** The correlation analysis of the demographic, lab, coeliac serology, and pathology parameters that significantly differ between the groups *p<0,05, **p<0,01 r: correlation coefficient; Co-eff: co-efficient; Neutrop: neutrophil; Lymphoc: lymphocyte; Anti-tTGA: anti-tissue transglutaminase antibody; Anti-EMA: anti-endomysium antibody;  H.pylori: Helicobacter pylori

Parameters	Co- eff.	Age	Gender (Male)	Anti-tTGA	Anti-EMA	H.pylori	Atrophy	Neutrop	Lymphoc
Age		1	,221*	0,103	-0,02	-0,067	,257*	-0,046	-0,064
			0,042	0,349	0,858	0,543	0,018	0,675	0,56
Gender (Male)		,221*	1	0,111	0,147	-0,108	0,144	-0,049	0,068
		0,042		0,312	0,18	0,327	0,189	0,657	0,539
Anti-tTGA	r	0,103	0,111	1	,679**	0,184	0,076	0,106	0,072
	p	0,349	0,312		0,000	0,093	0,491	0,335	0,512
Anti-EMA	r	-0,02	0,147	,679**	1	,248*	,226*	0,121	0,179
	p	0,858	0,18	0,000		0,022	0,038	0,269	0,101
Platelets	r	-0,059	-0,071	-0,131	-0,073	-0,06	-0,11	0,05	-0,007
	p	0,594	0,516	0,234	0,506	0,587	0,316	0,648	0,952
Lymphocytes	r	-0,192	-0,198	-0,142	0,052	0,046	-0,018	-0,049	0,04
	p	0,078	0,069	0,195	0,634	0,678	0,868	0,657	0,714
Urea	r	,438**	,273*	0,126	0,196	0,026	0,158	-0,062	-0,053
	p	0,000	0,011	0,25	0,072	0,813	0,149	0,57	0,631
Creatinine	r	0,209	,306**	-0,008	0,122	-0,046	0,134	-0,089	-0,06
	p	0,055	0,004	0,945	0,268	0,679	0,223	0,416	0,586
Albumin	r	,404**	-,224*	0,039	-,227*	-0,094	-,340**	-0,024	-0,191
	p	0,000	0,039	0,722	0,037	0,392	0,001	0,827	0,079

## Discussion

In this study, we aimed to identify the GI risk factors or etiologic factors for dyspepsia in patients with distal gastrectomy with respect to the normal population. We compared the dyspeptic patients with and without a history of distal gastrectomy and found some quite important results in our study. First of all, patients with distal gastrectomy had a higher prevalence of coeliac antibody positivity. Secondly, the patients with distal gastrectomy had a higher frequency of having HP as well as atrophy, lymphocyte, and neutrophil positivity in their gastric mucosa. And finally, the association of lower albumin levels with anti-EMA and atrophy positivity, as well as the association of anti-EMA positivity with HP, and atrophy positivity were revealed in patients with dyspepsia.

Although HP is a well-known risk factor for gastric neoplasms and eradication is recommended in patients who undergo distal gastrectomy for treatment of gastric neoplasia, some studies reported poor long-term outcomes in patients negative for HP infection after gastrectomy [10,11]. Moreover, an inverse relationship was reported between colonization of HP and gastroesophageal reflux disease, Barrett's esophagus, and esophageal adenocarcinoma [12]. Potential mechanisms for this association include decreased gastric acidity induced by long-term HP persistence, effects on gastric hormones, and microbiota colonizing the gastric and esophageal tissue [12]. However, the majority of the studies in the literature suggest a reduced risk of premalignant conditions and metachronous cancers with HP eradication in the remnant stomach [13]. Most of these studies were conducted in patients who underwent distal gastrectomy due to gastric carcinoma; whereas, our study was conducted with patients who had a history of antrectomy due to benign etiology. Similar to ours, Li et al. studied 281 patients with distal gastrectomy, excluding the ones with tumors, and revealed that HP infection exacerbates the severity of endoscopic remnant gastritis and chronic histological inflammation [14]. Even in our study consisting of a small number of patients, the prevalence of HP was significantly higher in patients with distal gastrectomy. When viewed from this aspect, screening and eradication of HP in dyspeptic patients with distal gastrectomy are crucial to prevent potential neoplasms.

The other interesting result of our study regarding HP was the correlation of anti-EMA with HP. Since HP infection and celiac disease, both can cause dyspeptic symptoms, a differential diagnosis should be made carefully. Particularly, chronic HP-associated duodenitis might cause diagnostic confusion since intraepithelial lymphocytes in the duodenal bulb may be present similar to CD [15]. Controversial studies exist in the literature regarding the association between CD and HP gastritis. Some studies reported a low frequency of CD as well as inflammatory bowel diseases (IBD) such as Crohn’s disease and ulcerative colitis in patients infected with HP [16-18]. Fujimori, in his editorial about this issue, suggests that this association might be caused by complex immunological relationships and host immune responses [19]. In a study conducted with 690 patients with CD, the authors reported a higher prevalence of HP in patients with treated CD and explained this association with the gluten-free diet and host immune response [20]. Similarly, a recent study conducted with 70 pediatric patients suggests that children with CD and HP infection had milder forms of enteropathy compared to children who are negative for HP [21]. Despite these studies, reviews analyzing the association of HP infection and CD in multiple articles reported otherwise. A review reported a few studies suggesting that HP infection may be the cause of the pathogenesis of lymphocytic gastritis in patients with CD, and its treatment may reduce the intestinal inflammation by reducing the number of intraepithelial lymphocytes, thus leading to an improvement of dyspeptic symptoms [22]. Another meta-analysis consisting of 25 papers and 141,355 participants showed that the HP infection rate of CD patients was 0.57 times greater compared to controls but the etiology remained uncertain [23]. Thus, our results that show a positive correlation between anti-EMA and HP would certainly contribute to the controversial literature. In addition to this, no studies exist analyzing the relationship between CD serological tests, one by one, with HP infection. This association should be analyzed by more studies with larger groups to identify the exact relationship between CD serology and HP. 

The correlation analysis also revealed a correlation between anti-EMA and atrophy positivity of the gastric specimens. Literature regarding the association of HP, CD, and atrophic gastritis is also controversial. A study evaluating the interaction between HP infection and untreated CD on gastric histological patterns revealed that in patients with HP infection, the untreated CD could represent a risk factor for follicular gastritis; however, it is associated with a lower prevalence of atrophic gastritis [24]. On the contrary, another study reported that a clinical presentation like atrophic gastritis is common in patients with CD [25]. None of these studies could support their evidence with pathophysiological mechanisms. Our hypothesis on this association is that a similar reaction to intraepithelial lymphocyte infiltration and subsequent villous atrophy of the duodenum might occur in the gastric mucosa in patients with CD. More studies are needed to clarify this relationship and the underlying pathophysiologic mechanism.

Another interesting result of our correlation analysis in our patients with dyspepsia was the negative correlation of serum albumin levels with anti-EMA and atrophy of the gastric mucosa. Atrophic gastritis may be identified as a severe form of gastritis since it was linked with long-standing HP infection and increased risk for gastric cancer, especially when extensive intestinal metaplasia is present [26]. In this aspect, it is comprehensible that serum albumin levels might be lower in the severe form of gastritis since it is a well-known negative acute phase reactant. Unfortunately, there are not many studies in the literature to determine whether albumin could be a prognostic factor for the progression of gastritis. The negative correlation of albumin with anti-EMA positivity could also be explained by the same hypothesis. Mean serum albumin concentrations were 4.4 mg/dl in the normal population while it was 3.0 mg/dl in patients with CD in a recent study conducted with the pediatric group [27]. In our study group, albumin levels were significantly lower in patients with distal gastrectomy. Since albumin is a poor prognostic factor for most of the inflammatory diseases and correlated with anti-EMA and atrophy positivity in our study group, these parameters might also be a poor prognostic factor for patients with distal gastrectomy. However, this hypothesis must also be evaluated by more studies with large populations. 

Plasma levels of platelets, lymphocytes, and albumin were found to be significantly lower, whereas urea, creatinine, and celiac antibody positivity were higher in patients with distal gastrectomy. Low platelets, lymphocytes, and albumin, as well as high urea and creatinine levels, are known to be important factors of mortality and morbidity in most diseases. For instance, a nationwide study consisting of 33,917 patients who underwent distal gastrectomy for treating gastric cancer revealed that levels of blood urea nitrogen >20 mg/dl, creatinine >1.2 mg/dl, albumin <3.8 mg/dl, platelets <12× 104/ml, white blood cells >9000/ml were associated with both morbidity and mortality [28]. Decreased levels of platelets, lymphocytes, and albumin might be caused by the chronic inflammatory process that constantly exists, and increased levels of kidney function tests might be caused by the impaired absorption of water due to restricted gastric mucosa in patients with distal gastrectomy.

The antrectomy group was significantly older and mostly consisted of males in our study. There were some reasons for this distribution. First of all, advanced age and male gender are famous risk factors for peptic ulcer diseases, and distal gastrectomy was the treatment of choice for these patients 30-40 years ago. Nowadays, even the complications of gastroduodenal ulcers, such as perforation and bleeding, are widely treated by minimally invasive techniques such as endoscopic interventions [3]. Thus, living without the distal part of their stomach has become a problem for the elderly. Since smoking rates are reported to be three times higher in males in Turkey and cigarette smoking is a well-known risk factor for peptic ulcer disease, there is no surprise in the discovery of male predominance of the patients with distal gastrectomy [29].

Although the age difference is statistically significant, the mean ages are approximately 55 for the control and 66 for the antrectomy group, which could not be considered a huge difference. But yet, age and gender differences between the groups are among the limitations of our study as well as being retrospective and consisting of a small study group. It would be better if we could add the time interval between the surgery date and the date of upper GI endoscopy to the analyses. Another limitation of our study is not having comorbidity status and drug history data in the files. Also, there was no data to indicate whether the gastrectomy patients were HP-positive or had CD prior to their gastrectomy. The fact that they have required an antrectomy rather than standard anti-ulcer treatment might indicate that they had been more complex and may have had other issues. They may have been resistant to HP eradication which could explain why they required surgery. Unfortunately, we did not have this information in the patient files. Studies with larger sample sizes are needed to clarify these potential theories for the increased incidence of HP and CD in the antrectomy group.

## Conclusions

Although distal gastrectomy is rarely preferred for the treatment of non-malignant gastroduodenal diseases, nowadays, clinicians still need to deal with patients with a history of distal gastrectomy. Since alterations in the physiological functioning and microbiological environment of the upper GI system are inevitable following the resection of the distal part of the stomach, clinicians should keep in mind the risk of increased incidence of HP and CD for these patients. However, randomized controlled trials are needed to understand the exact association and pathophysiological mechanism underlying the increased prevalence of HP and CD in patients with antrectomy.
